# Healthcare professionals’ knowledge, attitude and practice towards National Centralized Drug Procurement policy in central China: A cross-sectional study

**DOI:** 10.3389/fphar.2022.996824

**Published:** 2022-10-07

**Authors:** Anqi Huang, Xuanxuan Wang, Yun Tao, Likai Lin, Hong Cheng

**Affiliations:** ^1^ Department of Pharmacy, Zhongnan Hospital of Wuhan University, Wuhan, Hubei, China; ^2^ Hospital Management Institute of Wuhan University, Zhongnan Hospital of Wuhan University, Wuhan, Hubei, China

**Keywords:** knowledge, attitude, practice, National Centralized Drug Procurement policy, healthcare professionals

## Abstract

**Background:** National Centralized Drug Procurement (NCDP), an ongoing government-led policy starting in 2019 in China, aimed at reducing drug costs. During the implementation of NCDP, healthcare professionals (HCPs) still have a certain degree of concern about the policy, which affects the clinical use of related drugs.

**Objective:** This study aims to assess the level of knowledge, attitude and practice (KAP) of HCPs towards NCDP policy, together with the associated factors that determine their KAP.

**Methods:** A cross-sectional study was conducted between September and November of 2021 in 30 hospitals in Hubei province in Central China. A self-designed online questionnaire including KAP towards NCDP policy was administered to HCPs. Logistic regression analysis was adopted to identify the factors associated with KAP.

**Results:** A Total of, 742 HCPs completed the questionnaires. 43.4% of HCPs had good knowledge, 24.7% had a positive attitude, and 23.7% held good practice. Through multivariate logistic regression analysis, HCPs who are males, pharmacists, with senior professional titles and 6–10 years of professional working experience contributed to a higher knowledge level. Pharmacists and HCPs with good knowledge were more likely to have positive attitudes, while HCPs with higher education were less likely to have positive attitudes. Pharmacists, HCPs who had 11–20 years of professional working experience, worked in medium-size urban areas or had good knowledge were more likely to have good practice. Good practice is also associated with the positive attitude towards the efficacy of centralized-purchased medicines and impacts of NCDP policy.

**Conclusion:** Only a small percentage of HCPs had good KAP towards NCDP policy. Pharmacists showed better KAP than physicians. The positive attitude towards the efficacy of centralized-purchased medicines and impacts of NCDP policy contributed to better practice. High-quality clinical evidence on the therapeutic effects and safety of the centralized-purchased drugs is needed.

## Introduction

The fast rise of pharmaceutical expenditure and the resulting increase in total health expenses have brought a heavy burden to the finances and the daily medical care of the people around the world, accessibility and affordability of medicines have become major issues for healthcare systems globally (Seidman and Atun, 2017; Long et al., 2022; Wen et al., 2022; Zhang et al., 2022). And China is no exception (Ma et al., 2020). The pharmaceutical expenditure for each patient has risen from 36.59 yuan (5.33 dollars) to 467.04 yuan (67.98 dollars) between 1990 and 2008, with an annual growth rate of 15%, even higher than the GDP growth level (He et al., 2018). Curbing inflated drug prices has become the top priority of China’s medical reform. In recent years, from national negotiations to zero-price markups for drugs, to national centralized drug procurement (NCDP), the state has taken numbers of measures to alleviate the burden of medical care. Among them, NCDP policy plays a crucial role in reducing drug prices (Lan et al., 2022).

Actually, the centralized procurement model is not the first of its kind in China. Many countries from low-middle-income to high-income have adopted centralized procurement (or named “pooled procurement”) of medicines to control drug expenses (Dubois et al., 2021; Parmaksiz et al., 2022). For instance, in Denmark and Norway the respective central procurement agencies purchase the medicines for all public hospitals (Vogler et al., 2022). China has implemented NCDP policy by summarizing international experience. The NCDP policy led by government has started since 2019, which requires drug manufacturers to reduce the price of drugs and the government enters into contracts with manufacturers to purchase drugs for a certain amount of use, after which public healthcare institutions are required to enforce that price and amount of use (Chen et al., 2020). In 2019, the State took the lead in conducting a pilot project for centralized drug procurement, including four municipalities and seven sub-provincial cities (referred to as “4 + 7”) (Hua et al., 2021). 25 drugs including 22 generic drugs varieties which has passed consistency evaluation and 3 original research varieties were selected. Compared with the minimum purchase price of the same drug in the pilot cities in 2017, the selected varieties dropped by an average of 52%, and the highest drop was 96% (Yang et al., 2021). From the pilot to nationwide expansion and continuous operation, NCDP policy has been fully implemented across China. The sharp drop in selected drug prices has led to the price linkage of non-selective drugs (Wu et al., 2021), further promoting the price reduction effect, and effectively reduce patients’ medical burden, especially those with chronic and severe illnesses.

However, in the process of implementing NCDP policy, achievements and challenges coexist. Since the use of centralized-purchased drugs is ensured by the policy, the use of non-selected drugs is limited, which restricts physician’s prescription options and affects patients’ adherence. Moreover, due to lack of high-quality efficacy and safety evaluation of the centralized-purchased drugs, physicians still have a certain degree of concern about the policy (Dunne et al., 2014). And the extremely low price of the selected drugs makes patients doubt its quality and efficacy (Yang et al., 2021; Liang et al., 2022). In terms of the supply of selected drugs, there is a risk of supply shortages that affects the treatment of patients due to the drugs are only provided by one company. NCDP policy still needs to be continuously improved in practice.

Currently, there is little information about KAP regarding NCDP policy among HCPs. This may be an unexplored barrier to implement NCDP policy and reduce medical burden. In this study, we investigated KAP and the suggestions of HCPs towards the policy in Hubei province in Central China.

## Materials and methods

### Study design and participants

This was a cross-sectional study based on a questionnaire survey. This study was conducted among HCPs in 30 hospitals in Hubei Province in Central China between September and November of 2021.

### Sample size

To determine the sample size for this survey, we utilized the well-known online sample size calculator Raosoft (Raosoft , 2019; Memon et al., 2020). We set the margin of error to 4%, the confidence level at 95% (Marzan et al., 2021). Based on the official figure of 255,995 HCPs working in hospitals in Hubei Province in 2019 (China Government Statistics Information Center, 2020), the required sample size for this survey was 599.

### Inclusion and exclusion criteria

The inclusion criteria were HCPs working at hospitals in Hubei Province at the time the survey conducted. The questionnaire using screeners that automatically excluded any respondents whose filling time was less than 1 min or whose work location was not in Hubei Province.

### Questionnaire design

Three clinical pharmacists developed the KAP questionnaire after conducting extensive literature review and discussion. The content validity of the questionnaire was reviewed by three experts in the field (one expert from pharmacoepidemiology, one expert from health policy, and one expert from cardiology). Pilot questionnaires were administered to 37 HCPs in two hospitals and analyzed for reliability and validity. The content reliability of the scale has a Cronbach’s alpha of 0.781, indicating acceptable internal consistency (Taber, 2017). And Kaiser-Meyer-Olkin and Bartlett’s sphericity test revealed the KMO value was 0.854 (>0.6, *p* < 0.05), showing excellent validity (Sadowska et al., 2021).

The questionnaire was composed of 18 mandatory single-choice items and one multiple-choice item, and it consisted of five sections: 1) HCPs’ characteristics: profession, education, working experience, etc.; 2) Knowledge part: familiarity with NCDP policy and consistency evaluation of generic drugs, this part was provided on a 5-level Likert scale (1 = “very unfamiliar”, 2 = “less familiar”, 3 = “general”, 4 = “more familiar”, 5 = “very familiar”) ; 3) Attitude part: concerning about the policy impact, this part was provided on a 5-level Likert scale (1 = “strongly disagree”, 2 = “disagree”, 3 = “uncertain”, 4 = “agree”, 5 = “strongly agree”); 4) Practice part: interpretation, guidance for patients, and drug choice, this part was provided on a 5-level Likert scale, the options were assigned in order of 5 to 1 point according to the degree; 5) Suggestions towards NCDP policy: provided in multiple-choice form. The cut-off points for good (or positive) knowledge, attitude or practice was set at ≥ 80% of total points for the individual part (AlRasheed et al., 2021b). Detailed information related to KAP towards NCDP policy were presented in Supplementary Material.

### Data collection

A snowball sampling method was adopted for this study (Leighton et al., 2021). We created an online questionnaire using Questionnaire Star tool. The invitation to complete the questionnaire was distributed through WeChat groups and emails of HCPs. Participants were encouraged to distribute the questionnaire to as many colleagues as possible. The average time to complete the questionnaire was 15 min. Before starting the survey, each participant was asked to review the aim of the survey and to then fill out an electronic informed consent. Participants could withdraw from the survey at any time. We obtained 889 questionnaires by using the snowball method of distribution. 147 questionnaires were excluded based on the exclusion criteria. As a result, 742 questionnaires were evaluated in this study.

### Data processing

Data statistics were applied by using IBM SPSS Statistics 26. Descriptive analysis of frequencies and proportions of KAP levels was performed. The correlation between KAP levels and different demographic characteristics were evaluated through univariate logistic regression. The demographic factors with *p* < 0.1 in the univariate logistic regression analysis were taken as covariates in the multivariate logistic regression. The multivariate logistic regression method was conducted to identify the associated factors of KAP levels. The statistical significance level was set at *p* < 0.05.

## Result

### Demographic characteristics of participants

A total of 742 HCPs were studied. More than half of them were females (56.7%). Most participants were pharmacists (351, 47.3%) and physicians (322, 43.4%). 463 HCPs (62.4%) came from large metropolitan areas, and 570 HCPs (76.8%) worked at tertiary hospitals (Table 1).

**TABLE 1:** Demographic characteristics of the studied HCPs.

Variables	Number (N = 742)	Percentage (%)
Gender		
Male	321	43.3
Female	421	56.7
Profession		
Physician	322	43.4
Pharmacist	351	47.3
Others	69	9.3
Professional title^*^		
Primary	190	25.5
Intermediate	275	37.0
Senior	208	28.0
Education level		
Technical secondary school and junior college	89	12.0
Bachelor	347	46.8
Master or above	306	41.2
Professional working experience (years)		
≤5	166	22.4
6–10	162	21.8
11–20	163	22.0
21–30	177	23.9
More than 30	74	10.0
City		
Large metropolitan areas	463	62.4
Medium-size urban areas	159	21.4
Small urban areas	120	16.2
Hospital category		
Tertiary hospitals	570	76.8
Secondary hospitals	126	17.0
Primary hospitals	46	6.2

* means 69 participants had missing data on their professional title.

### Healthcare professionals’ knowledge of National Centralized Drug Procurement policy

**FIGURE 1:**
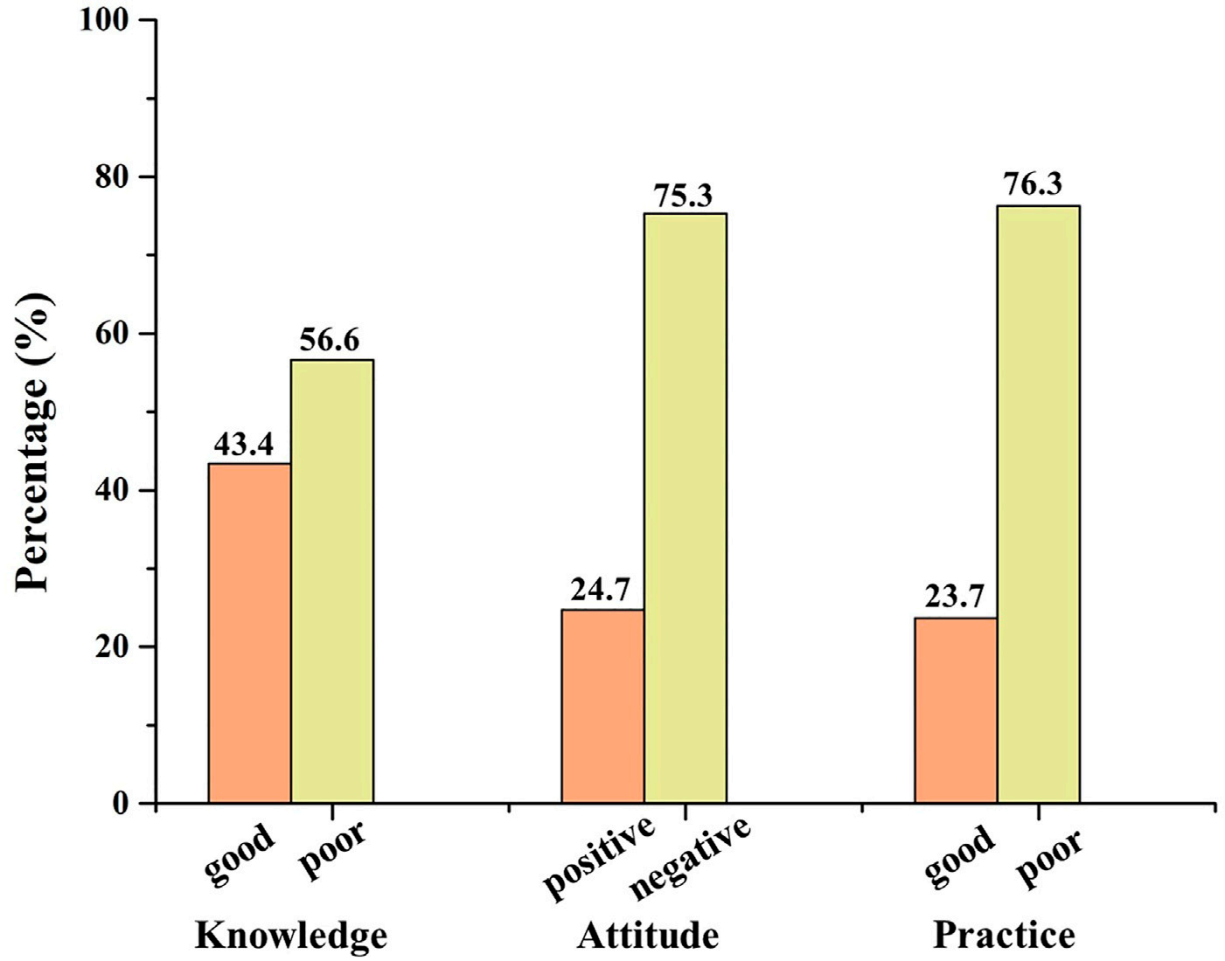
Knowledge, attitude, and practice of HCPs towards NCDP policy.

62.5% of HCPs agreed that they knew the policy of NCDP, and 47.9% of them agreed that they were familiar with the generic drug consistency evaluation (Supplementary Table S1). Overall, only 43.4% of HCPs got a good score for knowledge (Figure 1).

### Healthcare professionals’ attitude of National Centralized Drug Procurement policy

Although most participants agreed NCDP policy was reasonable and played a significant role in medical reform and reduce patients’ medication costs, merely 11.3% agreed there was no difference in quality or efficacy between brand-name drugs and generic drugs that had passed consistency evaluation, and only 44.7% agreed that the sharp drop in drug prices did not affect drug quality or efficacy. 49.4% of HCPs agreed NCDP policy can reduce patient-doctor disputes (Supplementary Table S2). On the whole, only 24.7% of HCPs had positive attitudes towards NCDP policy (Figure 1).

### Healthcare professionals’ practice of National Centralized Drug Procurement policy

24.3% of HCPs always or often explain NCDP policy to patients with doubts. Most HCPs (70.9%) always or frequently encourage patients to use centralized procurement medicines. Only 23.7% of HCPs always consider centralized procurement drugs for themselves or their families, while 37.9% sometimes do (Supplementary Table S3). Based on the practice score, only 23.7% of HCPs had good practice (Figure 1).

### Factors associated with knowledge level

Amongst the variables involved in the univariate analysis, gender, profession, professional title, professional working experience, and hospital grade have shown a significant association (*p* < 0.05) (Table 2). In the multivariate logistic analysis, pharmacists (OR = 3.30, 95% CI: 2.28–4.78), HCPs with senior professional titles (OR = 2.38, 95% CI: 1.30–4.33), and 6–10 professional working experience (OR = 1.86, 95% CI: 1.08–3.20) were more likely to have a good knowledge, while female HCPs (OR = 0.59, 95% CI: 0.42–0.82) tended to have poor knowledge.

**TABLE 2:** Univariable and multivariable analysis of associated factors of HCPs’ knowledge towards NCDP policy.

Demographic data	Knowledge		Unadjusted OR (95% Cl)	*p* value	Adjusted OR (95% Cl)	*p* value
	Poor, *N* (%)	Good, *N* (%)				
Gender						
Male	159 (37.9%)	162 (50.3%)	1		1	
Female	261 (62.1%)	160 (49.7%)	0.60 (0.45–0.81)	0.001*	0.59 (0.42–0.82)	0.002*
Profession						
Physician	212 (50.5%)	110 (34.2%)	1		1	
Pharmacist	153 (36.4%)	198 (61.5%)	2.49 (1.83–3.41)	<0.001*	3.30 (2.28–4.78)	<0.001*
Others	55 (13.1%)	14 (4.3%)	0.49 (0.26–0.92)	0.027*		
Professional title						
Primary	121 (33.2%)	69 (22.4%)	1		1	
Intermediate	144 (39.5%)	131 (42.5%)	1.60 (1.09–2.33)	0.016*	1.52 (0.96–2.41)	0.076
Senior	100 (27.4%)	108 (35.1%)	1.89 (1.27–2.83)	0.002*	2.38 (1.30–4.33)	0.005*
Education level						
Technical secondary school and junior college	50 (11.9%)	39 (12.1%)	1			
Bachelor	203 (48.3%)	144 (44.7%)	0.91 (0.57–1.46)	0.692		
Master or above	167 (39.8%)	139 (43.2%)	1.07 (0.66–1.72)	0.789		
Professional working experience (years)						
≤5	123 (29.3%)	43 (13.4%)	1		1	
6–10	88 (21.0%)	74 (23.0%)	2.41 (1.51–3.83)	<0.001*	1.86 (1.08–3.20)	0.026*
11–20	81 (19.3%)	82 (25.5%)	2.90 (1.82–4.60)	<0.001*	1.73 (0.96–3.11)	0.068
21–30	93 (22.1%)	84 (26.1%)	2.58 (1.64–4.08)	<0.001*	1.30 (0.69–2.47)	0.417
31–40	35 (8.3%)	39 (12.1%)	3.19 (1.80–5.66)	<0.001*	1.37 (0.64–2.94)	0.413
City						
Large metropolitan areas	256 (61.0%)	207 (64.3%)	1			
Medium-size urban areas	98 (23.3%)	61 (18.9%)	0.77 (0.53–1.11)	0.164		
Small urban areas	66 (15.7%)	54 (16.8%)	1.01 (0.68–1.52)	0.954		
Hospital grade						
Tertiary hospitals	333 (79.3%)	237 (73.6%)	1			
Secondary hospitals	61 (14.5%)	65 (20.2%)	1.50 (1.02–2.21)	0.041*	0.95 (0.61–1.49)	0.833
Primary hospitals	26 (6.2%)	20 (6.2%)	1.08 (0.59–1.98)	0.802	0.98 (0.43–2.23)	0.964

The bold values and * mean statistically significant data, mainly for ease of viewing.

### Factors associated with attitude level

In the univariate analysis, five factors were significantly correlated with a positive attitude level, which were the profession, education level, city, hospital grade and knowledge level (*p* < 0.05), as exhibited in Table 3. In the multivariate logistic analysis, pharmacists (OR = 1.97, 95% CI: 1.24–3.13) and HCPs who have good knowledge (OR = 5.46, 95% CI: 3.66–8.16) were more likely to have positive attitudes. However, HCPs with bachelor and above educational background were less likely to have positive attitudes (OR = 0.53, 95% CI: 0.30–0.95; OR = 0.37, 95% CI: 0.19–0.74).

**TABLE 3:** Univariable and multivariable analysis of associated factors of HCPs’ attitude towards NCDP policy.

Demographic data	Attitude		Unadjusted OR (95% Cl)	*p* value	Adjusted OR (95% Cl)	*p* value
	Negative, *N* (%)	Positive, *N* (%)				
Gender						
Male	246 (44.0%)	75 (41.0%)	1			
Female	313 (56.0%)	108 (59.0%)	1.13 (0.81–1.59)	0.474		
Profession						
Physician	265 (47.4%)	57 (31.1%)	1		1	
Pharmacist	250 (44.7%)	101 (55.2%)	1.88 (1.30–2.71)	0.001*	1.97 (1.24–3.13)	0.004*
Others	44 (7.9%)	25 (13.7%)	2.64 (1.50–4.66)	0.001*		
Professional title						
Primary	141 (27.4%)	49 (31.0%)	1			
Intermediate	208 (40.4%)	67 (42.4%)	0.93 (0.61–1.42)	0.727		
Senior	166 (32.2%)	42 (26.6%)	0.73 (0.46–1.16)	0.185		
Education level						
Technical secondary school and junior college	54 (9.7%)	35 (19.1%)	1		1	
Bachelor	257 (46.0%)	90 (49.2%)	0.54 (0.33–0.88)	0.013*	0.53 (0.30–0.95)	0.034*
Master or above	248 (44.4%)	58 (31.7%)	0.36 (0.22–0.60)	<0.001*	0.37 (0.19–0.74)	0.005*
Professional working experience (years)						
≤5	125 (22.4%)	41 (22.4%)	1			
6–10	118 (21.1%)	44 (24.0%)	1.14 (0.69–1.86)	0.611		
11–20	126 (22.5%)	37 (20.2%)	0.90 (0.54–1.49)	0.670		
21–30	136 (24.3%)	41 (22.4%)	0.92 (0.56–1.51)	0.739		
31–40	54 (9.7%)	20 (10.9%)	1.13 (0.61–2.11)	0.702		
City						
Large metropolitan areas	358 (64.0%)	105 (57.4%)	1		1	
Medium-size urban areas	119 (21.3%)	40 (21.9%)	1.15 (0.75–1.74)	0.524	1.13 (0.68–1.90)	0.636
Small urban areas	82 (14.7%)	38 (20.8%)	1.58 (1.02–2.46)	0.042*	1.60 (0.96–2.67)	0.071
Hospital grade						
Tertiary hospitals	441 (78.9%)	129 (70.5%)	1		1	
Secondary hospitals	87 (15.6%)	39 (21.3%)	1.53 (1.00–2.35)	0.049*	0.87 (0.51–1.49)	0.608
Primary hospitals	31 (5.5%)	15 (8.2%)	1.65 (0.87–3.16)	0.127	0.76 (0.34–1.70)	0.506
Knowledge						
Poor	366 (65.5%)	54 (29.5%)	1		1	
Good	193 (34.5%)	129 (70.5%)	4.53 (3.15–6.51)	<0.001*	5.46 (3.66–8.16)	<0.001*

The bold values and * mean statistically significant data, mainly for ease of viewing.

### Factors associated with practice level

In the univariate analysis, HCPs who are pharmacists, have 6–20 professional working years, work in medium-size urban areas, own good knowledge and attitude tend to hold better practice level (Table 4). After adjustment of covariates, the multivariate model revealed that pharmacists (OR = 2.34, 95% CI: 1.46–3.73) HCPs who have 11–20 professional working experience (OR = 2.37, 95% CI: 1.39–4.06), work in medium-sized urban areas (OR = 2.16, 95% CI: 1.34–3.49), or have good knowledge (OR = 2.98, 95% CI: 2.02–4.39) were more likely to have good practice. Besides, good practice was associated with several attitudes, such as agreement that there is no efficacy difference between original branded drugs and domestically generic drugs that have passed consistency evaluation (OR = 2.08, 95% CI: 1.21–3.56), NCDP policy can effectively reduce the healthcare burden of chronically ill patients (OR = 2.20, 95% CI: 1.19–4.06) and can effectively reduce the patient-doctor disputes (OR = 1.72, 95% CI: 1.16–2.57).

**TABLE 4:** Univariable and multivariable analysis of associated factors of HCPs’ practice towards NCDP policy.

Demographic data	Practice		Unadjusted OR (95% Cl)	*p* value	Adjusted OR (95% Cl)	*p* value
	Poor, *N* (%)	Good, *N* (%)				
Gender						
Male	236 (41.7%)	85 (48.3%)	1			
Female	330 (58.3%)	91 (51.7%)	0.77 (0.55–1.08)	0.123		
Profession						
Doctor	258 (45.6%)	64 (36.4%)	1		1	
Pharmacist	258 (45.6%)	93 (52.8%)	1.45 (1.01–2.09)	0.043*	2.34 (1.46–3.73)	<0.001*
Others	50 (8.8%)	19 (10.8%)	1.53 (0.85–2.78)	0.160		
Professional title						
Primary	154 (29.8%)	36 (22.9%)	1			
Intermediate	203 (39.3%)	72 (45.9%)	1.52 (0.97–2.38)	0.070		
Senior	159 (30.8%)	49 (31.2%)	1.32 (0.81–2.14)	0.263		
Education level						
Technical secondary school and junior college	67 (11.8%)	22 (12.5%)	1			
Bachelor	264 (46.6%)	83 (47.2%)	0.96 (0.56–1.65)	0.875		
Master or above	235 (41.5%)	71 (40.3%)	0.92 (0.53–1.60)	0.767		
Professional working experience (years)						
≤5	138 (24.4%)	28 (15.9%)	1		1	
6–10	118 (20.8%)	44 (25.0%)	1.84 (1.08–3.13)	0.025*	1.66 (0.97–2.86)	0.066
11–20	113 (20.0%)	50 (28.4%)	2.18 (1.29–3.69)	0.004*	2.37 (1.39–4.06)	0.002*
21–30	138 (24.4%)	39 (22.2%)	1.39 (0.81–2.39)	0.229	1.42 (0.82–2.47)	0.214
31–40	59 (10.4%)	15 (8.5%)	1.25 (0.62–2.52)	0.526	1.23 (0.60–2.51)	0.574
City						
Large metropolitan areas	363 (64.1%)	100 (56.8%)	1		1	
Medium-size urban areas	110 (19.4%)	49 (27.8%)	1.62 (1.08–2.42)	0.019*	2.16 (1.34–3.49)	0.002*
Small urban areas	93 (16.4%)	27 (15.3%)	1.05 (0.65–1.71)	0.831	1.13 (0.69–1.85)	0.622
Hospital grade						
Tertiary hospitals	432 (76.3%)	138 (78.4%)	1			
Secondary hospitals	98 (17.3%)	28 (15.9%)	0.89 (0.56–1.42)	0.636		
Primary hospitals	36 (6.4%)	10 (5.7%)	0.87 (0.42–1.80)	0.706		
Knowledge						
Poor	366 (64.7%)	54 (30.7%)	1		1	
Good	200 (35.3%)	122 (69.3%)	4.13 (2.87–5.95)	<0.001*	2.98 (2.02–4.39)	<0.001*
NCDP policy has played a big role in the medical reform						
Uncertain, disagree and strongly disagree	75 (13.3%)	7 (4.0%)	1		1	
Agree and strongly agree	491 (86.7%)	169 (96.0%)	3.69 (1.67–8.16)	0.001*	1.21 (0.49–2.97)	0.680
The country’s policy orientation of reducing drug prices and medical costs through NCDP policy is reasonable						
Uncertain, disagree and strongly disagree	133 (23.5%)	12 (6.8%)	1		1	
Agree and strongly agree	433 (76.5%)	164 (93.2%)	4.20 (2.26–7.78)	<0.001*	1.59 (0.78–3.24)	0.198
The sharp reduction in drug prices (50%–96%) has no impact on the quality/efficacy of drugs						
Uncertain, disagree and strongly disagree	343 (60.6%)	67 (38.1%)	1		1	
Agree and strongly agree	223 (39.4%)	109 (61.9%)	2.50 (1.77–3.54)	<0.001*	1.16 (0.77–1.76)	0.486
There is no difference in the quality/efficacy of imported original research drugs and domestically generic drugs that have passed consistency evaluation.						
Uncertain, disagree and strongly disagree	526 (92.9%)	132 (75.0%)	1		1	
Agree and strongly agree	40 (7.1%)	44 (25.0%)	4.38 (2.74–7.01)	<0.001*	2.08 (1.21–3.56)	0.008*
NCDP policy is effective in reducing the medical burden of patients with chronic diseases						
Uncertain, disagree and strongly disagree	170 (30.0%)	16 (9.1%)	1		1	
Agree and strongly agree	396 (70.0%)	160 (90.9%)	4.29 (2.49–7.40)	<0.001*	2.20 (1.19–4.06)	0.012*
NCDP policy can effectively decrease the patient-doctor disputes						
Uncertain, disagree and strongly disagree	319 (56.4%)	56 (31.8%)	1		1	
Agree and strongly agree	247 (43.6%)	120 (68.2%)	2.77 (1.94–3.96)	<0.001*	1.72 (1.16–2.57)	0.008*

The bold values and * mean statistically significant data, mainly for ease of viewing.

### Healthcare professionals’ comments and suggestions on National Centralized Drug Procurement policy

The first three issues on NCDP policy, according to HCPs, were patients’ doubts about the efficacy of centralized procurement medicines (74.1%), the limited prescribing options of physicians (47.0%), and the unsatisfactory treatment outcomes of the selected drugs (40.6%) (Supplementary Table S4). Another 136 HCPs provided other suggestions, as seen in Table 5.

**TABLE 5:** Other suggestions from 136 HCPs on NCDP policy.

Suggestions	Number of HCPs
Ⅰ The efficacy and safety of centralized-purchased medicines need to be guaranteed.	42
Ⅱ Drug prices should be set scientifically, and reasonable profit margins should be retained for enterprises to encourage them to develop and improve innovative drugs.	19
Ⅲ Improve the choice freedom of original research drugs or centralized-purchased drugs.	26
Ⅳ The medical insurance catalogue need update with the centralized-purchased drugs catalogue, and the medical insurance payment standards need to be optimized.	6
Ⅴ The salary of medical staff and the level of hospital information construction should be ameliorated.	12
Ⅵ Varieties of centralized-purchased drugs need to be further optimized.	19
Ⅶ Strengthen the propaganda of consistent evaluation generic drugs and NCDP policy, and increase the recognition of medical staff and patients in centralized-purchased drugs.	8
Ⅷ Optimize the procurement methods, time, and supply channels of the drugs.	12

## Discussion

As the main body of the execution of NCDP policy, behaviors of HCPs are the key to put the policy into effect in medical institutions. It has been reported the increase in knowledge level will change the attitudes, and in turn change the related practices, showing the consistency of KAP (Kasemy et al., 2020; Iradukunda et al., 2021; Rahmah et al., 2021). Thus, this cross-sectional study aims to evaluate KAP level and associated factors of HCPs towards NCDP policy, which is critical to reflect the implementation of the policy in medical institutions and identify potential resistance.

In this study, it showed that 37.5% of HCPs are unclear with NCDP policy, and 52.1% have little knowledge about generic drug consistency evaluation, which is in line with the previous report about poor knowledge of health practitioners in Palestine on generic medicine products (Shraim et al., 2017). Pharmacists have a better understanding about NCDP policy compared to doctors, probably because of pharmacists’ greater exposure and sensitivity to the policy related to drugs. HCPs with senior professional title and longer working years had greater knowledge, which can be explained by their easier access to relevant knowledge and richer work experience.

The positive attitude of HCPs is considered as an impetus to carrying NCDP policy out. This study showed that due to the huge price cut for centralized procurement drugs, most HCPs agree NCDP policy is important, reasonable and useful to release the burden on patients; nearly half believe the policy can ease the doctor-patient relationship. For instance, the monthly cost of entecavir (a drug for treating chronic hepatitis B) for patients was as high as 400–500 yuan before NCDP policy; at present, the cost has been reduced to 49.8 yuan per month (Zhan et al., 2021). The reduction of medication cost also improved patient adherence (Dietze et al., 2020). However, it is worth noting that 88.7% of HCPs do not know or consider there exists big differences in the therapeutic effect between generic and branded drugs. This is consistent with the surveys that quite a few HCPs expressed negative perceptions about the efficacy of generic drugs (Shrank et al., 2011). Since the current consistency evaluation of generic drugs only involves the pharmaceutical characteristics and bioequivalence (Hansen et al., 2017; Chazin et al., 2019), such attitude might be related to lack of high-quality clinical evidence of generic drugs about their comparative safety and effectiveness relative to their brand-name counterparts (Choudhry et al., 2016; Zeng and Song, 2019), especially in the real world. Consequently, 55.2% of HCPs do not know or disagree that the quality of centralized-purchased medicines, mostly generic drugs, will not be affected by price cuts, indicating that they were concerned about the quality and efficacy of centralized-purchased drugs, which may become a major barrier to advancing NCDP policy (Kesselheim et al., 2016; Tang et al., 2019). Pharmacists have more positive attitudes, which can be ascribed to their better knowledge, which is in accordance with the results of other similar researches (AlRasheed et al., 2021a). Notably, HCPs with higher education background have poorer attitudes towards the policy, which may because they are more aware that the curative effect of generic drugs not only depends on consistency evaluation, especially for narrow therapeutic index drugs (Qu et al., 2021), but also should be supported by strong clinical comprehensive evaluation.

Our findings indicate that practice levels among HCPs towards NCDP policy are unsatisfactory. Only a small number of HCPs often or always interpreted the policy for patients, possibly due to their short of understanding and confidence in the quality and efficacy of generic drugs (Dunne et al., 2014). However, when patients refused to use centralized-purchased drugs, the majority of HCPs encouraged patients to accept it, which shows the cooperation of HCPs with the policy. State Council issued official requirements that hospitals should make procurement plans based on no less than 80% of the actual use of the same generic drugs in the previous year, which also put pressure on HCPs to encourage the use of centralized-purchased drugs (China State Council, 2015; Wang et al., 2021). However, quite a few HCPs do not consider centralized-purchased drugs when choosing medicines for themselves and their families, which further suggests their limited confidence in the efficacy of centralized-purchased drugs (Gao et al., 2021). Besides, there is a significant association between HCPs’ characteristics and practice levels. Pharmacists still showed better practice, supported by their better knowledge and attitude towards the policy, which is in keeping with the study that cognition and attitudes affect the behaviors and coping strategies (Zhang et al., 2020). The difference of knowledge level has great significance on HCPs’ practice level, which indicates education intervention is one strategy for better practice (Gidey et al., 2020; Hu et al., 2022). Certain attitudes of HCPs also have a great impact on behavior: HCPs who agree that NCDP policy can reduce the burden of chronic disease patients and alleviate the contradiction between doctors and patients have higher degree of satisfaction to the policy and are more inclined to have better practice. More importantly, HCPs who believe differences in the quality between centralized-purchased medicines and brand-name medicines are more likely to have poorer practices, which shows the necessity of providing clinical evidences for the treatment outcome of the centralized-purchased drugs and thereby enhancing the endogenous motivation of HCPs to use the drugs.

Our study is helpful for understanding the KAP of HCPs, exploring the possible barriers towards NCDP policy and improving the policy. First, we found that most HCPs showed relatively poor KAP towards NCDP policy, though it has been implemented for around 2 years. Second, our research showed that the key factor lowering KAP is HCPs’ suspicion on the quality of centralized-purchased drugs. Thus, high-quality research is required to provide enough clinical evidence for the effectiveness and safety of generic medicines, which is critical to enhance HCP’s understanding and confidence for using centralized-purchased medicines. Third, these findings have important implications for completion of procurement volume in the medical institutions as well as the function of NCDP policy in reducing drug prices and increasing drug accessibility. Besides, in the context of the continuous rise in global drug expenditure, it can also provide experience and reference for other countries or regions that need to control drug costs.

## Strengths and limitations

Our study has several strengths. First, we collected adequate participant samples with different working status from 30 healthcare institutions. Second, the timing of our investigation is reasonable, when NCDP policy has been implemented for around 2 years, and KAP of HCPs towards it can be fully exposed. Third, to our knowledge, this is the first survey that investigated KAP level of HCPs towards NCDP policy in China.

Our study also has some limitations. First, this study only involves HCPs from tertiary and secondary hospitals in large and medium cities in Central China, therefore the data could not be generalizable to primary hospitals or hospitals in rural areas. Second, the participants mainly include pharmacists and physicians, which could not fully show all types of HCPs’ KAP levels towards the policy.

## Conclusion

Only a small percentage of HCPs had good knowledge, attitudes and practice towards NCDP policy. Overall, pharmacists showed better understanding, attitudes and practice than physicians. Good practice was associated with the positive attitudes towards the efficacy of centralized-purchased medicines and impacts of NCDP policy. High-quality clinical evidences on the therapeutic effects and safety of the centralized-purchased drugs is needed.

## Data Availability

The original contributions presented in the study are included in the article/Supplementary Material, further inquiries can be directed to the corresponding authors.
