# Supplementary material for: Healthcare professionals’ knowledge, attitude and practice towards National Centralized Drug Procurement policy in central China: A cross-sectional study
Source: Front Pharmacol. 2022 Oct 7;13:996824. doi: 10.3389/fphar.2022.996824 (PMC9585188; doi:10.3389/fphar.2022.996824)
Supplement: Supplementary file 1 [file DataSheet1.pdf]

Supplementary Material

1 Supplementary Tables

Table S1 Knowledge of the studied groups (N=742) about NCDP policy.

Knowledge	Number	%
1. Have you known about national centralized drug procurement (NCDP) policy?		
Very familiar	135	18.2
More familiar	329	44.3
General	182	24.5
Less familiar	65	8.8
Very unfamiliar	31	4.2
2. Have you known about consistency evaluations of generic drugs?		
Very familiar	89	12.0
More familiar	266	35.9
General	241	32.5
Less familiar	70	9.4
Very unfamiliar	76	10.2

Table S2 Attitude of the studied groups (N=742) about NCDP policy.

Attitude	Number	%
1. NCDP policy has played a big role in the medical reform.		
Strongly agree	160	21.6
Agree	500	67.4
Uncertain	15	2.0
Disagree	60	8.1
Strongly disagree	7	0.9
2. The country's policy orientation of reducing drug prices and reducing medical expenses through NCDP policy is reasonable.		
Strongly agree	237	31.9
Agree	360	48.5
Uncertain	109	14.7
Disagree	29	3.9
Strongly disagree	7	0.9
3. There is no difference in the quality/efficacy of imported original research drugs and domestically generic drugs that have passed consistency evaluation.		
Strongly agree	27	3.6
Agree	57	7.7
Uncertain	81	10.9
Disagree	557	75.1
Strongly disagree	20	2.7
4. The sharp reduction in drug prices (50%-96%) has no impact on the quality/efficacy of drugs.		
Strongly agree	113	15.2
Agree	219	29.5
Uncertain	159	21.4
Disagree	113	15.2
Strongly disagree	138	18.6
5. NCDP policy is effective in reducing the medical burden of patients with chronic diseases.		
Strongly agree	236	31.8
Agree	320	43.1
Uncertain	165	22.2
Disagree	13	1.8
Strongly disagree	8	1.1
6. NCDP policy can effectively improve the patient-doctor disputes.		
Strongly agree	99	13.3
Agree	268	36.1
Uncertain	240	32.4
Disagree	117	15.8
Strongly disagree	18	2.4

Table S3 Practice of the studied groups (N=742) about NCDP policy.

Practice	Number	%
1. Did you interpret patient consultation about NCDP policy?		
Always	36	4.9
Often	144	19.4
Sometimes	282	38.0
Rarely	143	19.3
Never	137	18.5
2. When the patients refused to accept the prescriptions containing centralized procurement drugs, would you guide the patients to use centralized procurement drugs?		
Always	13	1.8
Often	513	69.1
Uncertain	62	8.4
Rarely	148	20.0
Never	6	0.8
3. Do you and your family choose centralized procurement drugs for daily medication?		
Always	176	23.7
Sometimes	281	37.9
Random	183	24.7
Rarely	85	11.5
Never	17	2.3

Table S4 Comments of HCPs on NCDP policy (one multiple-choice).

What do you think are the problems of NCDP policy?	Number	%
The restricted prescription right, the inconvenient daily diagnosis and treatment	349	47.0
The unsatisfactory drug quality, unsatisfactory diagnosis and treatment effect	301	40.6
Patients' disapproval of the quality/efficacy of centralized-purchased drugs affects normal diagnosis and treatment work	550	74.1
No problems	71	9.6
Others	136	18.3
